# Terms, Definitions, Nomenclature, and Routes of Fluid Administration

**DOI:** 10.3389/fvets.2020.591218

**Published:** 2021-01-15

**Authors:** Rosalind S. Chow

**Affiliations:** Department of Veterinary Clinical Sciences, College of Veterinary Medicine, University of Minnesota, St. Paul, MI, United States

**Keywords:** intraosseous, subcutaneous, intravenous, terminology, rehydration, fluid therapy, glossary, fluid administration methods

## Abstract

Fluid therapy is administered to veterinary patients in order to improve hemodynamics, replace deficits, and maintain hydration. The gradual expansion of medical knowledge and research in this field has led to a proliferation of terms related to fluid products, fluid delivery and body fluid distribution. Consistency in the use of terminology enables precise and effective communication in clinical and research settings. This article provides an alphabetical glossary of important terms and common definitions in the human and veterinary literature. It also summarizes the common routes of fluid administration in small and large animal species.

## Introduction

Fluid therapy is an important component in the treatment of many hospitalized veterinary patients. The breadth of literature on fluid therapy-related concepts and management strategies is continually expanding and clarity in the use of the terminology is essential for effective communication and patient care. Using incorrect terms can lead to misunderstanding, misinterpretations, and inappropriate therapeutic decisions. The first aim of this article is to provide a glossary of key terms with reference to definitions found in the veterinary and human medical literature. Commonly used abbreviations, synonyms, and related words are listed after the associated term, where applicable. An expanded definition is provided for any term for which there is no widely accepted definition. The second aim of this article is to describe the different routes of fluid administration in veterinary species.

## Methods

For the glossary, a list of fluid therapy related terms was compiled based on common terms encountered in the fluid therapy literature pertaining to intravenous fluid classifications, intravenous fluid administration strategies, body fluid volume and hydration, body fluid compartments, body fluid composition, and acid-base balance. A systematic PubMed/MEDLINE search and literature review of the veterinary and human medical literature was performed between January to October 2020 to search for individual terms in the title, key words or abstract. The full glossary term was used as the search term by itself, individually as well as with the addition of the following words: “definition,” “veterinary,” and if needed, included terms denoting to specific veterinary species such as “dog,” “cat,” or “horse.”. For example, the search strategy for “Acute Normovolemic Hemodilution” used the following search terms: “acute normovolemic hemodilution,” “acute normovolemic hemodilution definition,” “acute normovolemic hemodilution veterinary,” “acute normovolemic hemodilution dog” and “acute normovolemic hemodilution cat.” Publications were considered if they were available in English and were published or in press. A minimum of 3 articles were reviewed for each search term, up to a maximum of 6 articles. Priority was given to articles from journals with a high impact factor, veterinary journals, and consensus-type articles, as well as the most recently published definitions. Case reports, comments, editorials and letters to the editor were excluded because they rarely contained definitions. The full text of all identified articles were screened for definitions to one or more glossary terms and multiple articles were evaluated in order to determine the degree of agreement. If the literature search revealed inconsistencies in the definition for any particular term, the literature search was expanded, and the most commonly encountered definition was provided with the addition of a notation mentioning the lack of widespread consensus. Where multiple similar terms could be used describe the same concept, the most frequently encountered term was listed in the glossary and synonyms were provided at the end of the applicable definition.

## Results

### Glossary of Terms

#### Absolute Hypovolemia

A reduction in total circulating blood volume. Absolute hypovolemia can be caused by dehydration (i.e., water and electrolyte loss) or the loss of blood from the body or into a body cavity (e.g., abdomen) (1, 2).

#### Acid

A substance that is capable of increasing the concentration of hydrogen ions (H^+^) when dissolved in water (aqueous solution) (3).

#### Acidemia

A blood pH that is below the normal physiologic range for the species in question (4).

#### Acidosis

A process in which there is a net accumulation of acid in the body (3).

#### Acute Normovolemic Hemodilution

A blood conservation strategy where a specific volume of whole blood is removed from the patient and stored, and replaced by sufficient volumes of crystalloid or colloid solutions to restore intravascular volume prior to surgery. The rationale of this technique is to reduce the loss of red blood cells from surgical bleeding through hemodilution, thereby lowering the need for allogenic blood transfusions. The reserved blood is subsequently returned to the patient during or after surgery (5). Synonym: *isovolemic hemodilution*.

#### Albumin

Circulating blood protein weighing 69 kDa that is synthesized by the liver and is the major determinant of plasma oncotic pressure (6).

#### Alkalemia

A blood pH that is above the normal physiologic range for the species in question (4).

#### Alkalosis

A process in which there is a net accumulation of alkali in the body (7).

#### Anion

A negatively charged atom or molecule, such as chloride (Cl^−^) and bicarbonate (HCO3^−^) (8).

#### Anion Gap (AG)

The calculated difference between the principle cations and anions in plasma. Anion gap is calculated by the following formula: AG = (Na^+^ + K^+^) − (Cl^−^ + HCO3^−^). Anion gap is useful to help narrow down the potential causes of metabolic acidosis (4, 8).

#### Autotransfusion

A blood conservation strategy used during hemorrhage or surgery where shed blood is collected, typically mixed with anticoagulant, filtered and reinfused into the patient (9). In human medicine, where it is typical to wash red blood cells prior to readministration, this process is also referred to as *blood salvage* or *cell salvage*.

#### Balanced Component Resuscitation

A fluid resuscitation strategy in severe trauma management where blood products are transfused in proportions similar to blood (1:1:1 ratio for packed red blood cells, plasma and platelets) (10, 11). Synonym: *balanced resuscitation*.

#### Balanced Crystalloid Solution

A fluid that contains an electrolyte composition (particularly sodium, potassium, and chloride) that is similar to that found in plasma. A balanced solution should maintain or normalize acid-base balance and be isosmotic and isotonic (i.e., not induce inappropriate fluid shifts) with normal plasma (4, 12, 13). Synonyms: *balanced isotonic electrolyte solution, polyionic crystalloid solution*, or *balanced salt solution*.

#### Base

A substance that is capable of accepting a hydrogen ion (H^+^) when dissolved in water (3).

#### Base Deficit

The amount of a strong base that must be added *in vitro* to 1 L of oxygenated blood to return the pH to 7.40, at a partial pressure of carbon dioxide of 40 mmHg, and temperature of 37°C, in the presence of metabolic acidosis (14). Represents a deficiency of base, or the negative form of base excess, where a BD of +1 mmol/L is equivalent to a BE of −1 mmol/L.

#### Base Excess

The amount of a strong acid in mmol/L that must be added *in vitro* to 1 L of oxygenated blood to return the pH to 7.40, at a partial pressure of carbon dioxide of 40 mmHg, and temperature of 37°C, in the presence of metabolic alkalosis (14). See also *standard base excess*.

#### Blood Volume

The total volume of blood contained within the circulatory system (15). Synonym: *vascular volume, intravascular volume*.

#### Buffered Crystalloid Solution

An intravenous fluid containing an acid-base buffer in order to help maintain or restore physiologic pH. This consists of an aqueous solution containing a mixture of electrolytes and a weak acid and its conjugate base. The most common buffers are bicarbonate or organic anions (e.g., lactate, acetate, gluconate) (4).

#### Cation

A positively charged atom (e.g., Na^+^, K^+^, Ca^2+^) or molecule (${\text{NH}}_{4}^{+}$) (7, 8).

#### Cell Salvage

See *autotransfusion*.

#### Central Venous Pressure (CVP)

Measurement of venous blood pressure within a large central vein, or more specifically, the cranial or caudal vena cava (16). A controversial method of assessing right ventricular preload, CVP is not correlated with total blood volume and it is not a good general predictor of fluid responsiveness (17, 18).

#### Colligative Properties

Alterations in the properties of a solvent due to the addition of solutes. Colligative properties depend on the concentration of molecules in solution, rather than the type of chemical species present (19, 20). Examples include vapor pressure, boiling point, freezing point, and osmotic pressure.

#### Colloid

Large molecular weight molecules (>30 kDa) that are preferentially retained in the intravascular space following intravenous administration. Types of natural colloids include plasma and albumin. Synthetic colloids include hydroxyethyl starches, dextrans, and gelatins (7, 21).

#### Colloid Osmotic Pressure

The osmotic force generated by large molecules (colloids) in solution when separated by a semipermeable membrane from a region with a different colloid concentration (22). The colloid osmotic pressure provided by plasma proteins is also referred to as *oncotic pressure*.

#### Colloid Solution

An intravenous fluid containing macromolecules dispersed in a crystalloid solution. It is administered to support intravascular volume or raise plasma colloid osmotic pressure (23–25). Natural colloid solutions include blood products and albumin. Synthetic (or artificial) colloid solutions include hydroxyethyl starches, dextrans and gelatins. Colloid fluid therapy is also referred to as *biophysical therapy* (26).

#### Constant Rate Infusion (CRI)

Continuous intravenous administration of a medication in order to maintain a steady delivery or plasma concentration (27).

#### Critical Hematocrit

The minimum hematocrit that supports adequate tissue oxygenation and below which organ ischemia will develop (28, 29).

#### Crystalloid

A solution that contains electrolytes and other small water soluble molecules, and/or dextrose. Crystalloids are categorized by their tonicity relative to plasma: isotonic, hypotonic, and hypertonic (23–25).

#### Cumulative Fluid Balance

The difference between all fluid inputs and outputs over a defined period of time (30, 31). Cumulative fluid balance over a 24-h period is also referred to as *daily fluid balance*.

#### Daily Fluid Balance

The difference between all fluids inputs and outputs during a 24-h period, generally excluding insensible losses (30, 31). Daily fluid balance can be negative, neutral or positive.

#### Damage Control Resuscitation

A resuscitation strategy used to treat severely traumatized patients in order to reduce the development of the “lethal triad” of hypothermia, coagulopathy and acidosis (32, 33). The key principles are the early use of blood products, avoidance of excessive crystalloid infusions which can cause dilutional coagulopathy, and permissive hypotension (32, 33).

#### Deescalation

Reduction of fluid administration due to clinical improvement of the patient (31).

#### Dehydration

The loss of body water, with or without salt, at a rate greater than the body can replace it (34–36).

#### Deresuscitation

Correcting fluid overload by using dialysis or diuretics to remove excess fluid (37, 38).

#### Early Goal-Directed Therapy

A protocol-driven treatment algorithm that aims to guide fluid, vasopressor, and other resuscitation therapy toward specific hemodynamic end-points, with the goal of optimizing oxygen delivery (24, 39). Originally developed for the treatment of patients with sepsis (39), the concept has since been adopted for the treatment of critically ill patients with other conditions as well as in perioperative settings (40). “Early” refers to its use during the initial stabilization period, otherwise it is described as *goal-directed hemodynamic therapy*.

#### Edema

Clinical manifestation of fluid accumulation within the interstitial tissue space (interstitial edema) or within cells (cellular edema) (41, 42).

#### Effective Osmole

An electrolyte (ion) that exerts an osmotic force (i.e., pull) across a semi-permeable membrane. Effective osmoles determine a solution's tonicity. Sodium ion is the predominant effective osmole in the body (43).

#### Electrolyte

Dissolved ions in solution that carry a positive or negative electric charge, such as sodium, potassium, chloride, and calcium (35).

#### Endothelial Glycocalyx (EG)

A negatively charged, mesh-like layer on the luminal surface of vascular endothelial cells, composed of membrane-bound glycoproteins and proteoglycans, which have an important role in regulating vascular permeability, endothelial anticoagulation and modulating interactions between the endothelium and the vascular environment (9, 44–46). EG damage and breakdown are particularly susceptible to fluid overload, catecholamine-induced damage and shock (46).

#### Euvolemia

Normal circulating blood volume (35, 47).

#### Fluid Administration

Delivery of fluids to a patient through an enteral or parenteral route (48). The rate of fluid administration should be described in ml/kg/min or ml/kg/h. Fluid rates described in ml/h or ml/min are meaningless unless they are referenced to body weight or body surface area. The duration of infusion should be stated in order to determine the total volume of fluid administered.

#### Fluid Balance

The net difference between bodily fluid gains or inputs (including enteral, subcutaneous and intravenous fluids, injectable medications, and blood products) and fluid losses or outputs (including urinary, gastrointestinal, blood losses) over a specified time period (49, 50). Insensible losses are usually omitted (30). Fluid gains exceeding fluid losses represents a positive fluid balance, whereas fluid losses exceeding gains lead to a negative fluid balance. Fluid balance can be expressed as a volume or as a percentage of body weight. Related terms: *daily fluid balance, cumulative fluid balance*.

#### Fluid Bolus

Rapid intravenous administration of a small or large volume of fluid for the purpose of restoring tissue perfusion, such as during the treatment of hypovolemic shock (31, 51–53).

#### Fluid Challenge

Rapid intravenous administration of a modest volume of fluid, usually a crystalloid, in order to assess the likelihood of volume-responsiveness in a patient with hemodynamic instability, while minimizing the risk of fluid overload (31, 54). A fluid challenge is typically followed by a fluid bolus in patients that exhibit a positive response to the fluid challenge.

#### Fluid Compartments

Describes the distribution pattern of total body fluid within several well-defined spaces separated from each other by cell membranes (55). Together, the intravascular and interstitial fluid compartments comprise the extra-cellular fluid space and contain approximately one-third of the total body water, while intracellular fluid compartment contains approximately two-thirds of the total body water (55).

#### Fluid Creep

This term has two different situation-dependent meanings. Fluid creep describes the administration of IV fluid to burn patients in excess of fluid requirements calculated by the Parkland Formula (56, 57). This is done in an effort to optimize hemodynamic status but may increase the risk of edema formation. Fluid creep also refers to the unintentional and unmeasured fluid volumes administered in the process of delivering medication and nutrition through enteral and parenteral routes during maintenance fluid therapy or undocumented oral fluid intake (58).

#### Fluid Infusion

Intravenous fluid administration (31).

#### Fluid Overload

An increase in total body fluid (typically both water and electrolytes) in excess of physiologic requirements. Some publications define it as a 10% or more increase in total body weight due to fluid administration which represents the threshold for an increased risk of adverse clinical effects such as pulmonary edema, peripheral edema or body cavity effusion (30, 31, 59–61). This non-specific term is loosely used and in some cases may be replaced by more specific concepts, such as *volume overload*, which refers to excess fluid in the intravascular fluid compartment, or *overhydration*, which describes excessive pure water gain in the body.

#### Fluid Responsiveness

The ability for hemodynamic parameters to improve in response to a fluid challenge or bolus (51, 62). There is some variability in the specific parameters and magnitude of change that are considered consistent with a positive response. However, many definitions consolidate around an increase in cardiac output (51, 62) or stroke volume (52, 54) by at least 10–15% from baseline following administration of a fluid bolus that is delivered over <15 (51, 52) or 30 min (53). Others consider a patient to be fluid responsive if there is a significant change in one or more of the following: increase in mean arterial pressure of more than 10 mm Hg, decrease in heart rate of more than 10 BPM, increase in central venous pressure (>2 cm H_2_O) or an increase urine output (53, 63).

#### Fluid Resuscitation

The administration of intravenous fluids to reverse life-threatening tissue hypoperfusion (31, 62). Synonym: *volume resuscitation*.

#### Fluid Retention

An increase in net fluid balance resulting accumulation of excess fluids in body tissues and weight gain (64–66) and in some cases, peripheral edema (66). This is due to physiological or pathological processes promoting renal reabsorption and fluid conservation, such as during dehydration, pregnancy, anesthesia, acute kidney injury (AKI) and congestive heart failure (64).

#### Fluid Therapy

The unnatural process of administering fluids as a treatment or preventative measure to maintain or restore normal body fluid balance.

#### Fluid Titration

Adjustment of intravenous fluid administration choice, rate, volume and timing in order to improve hemodynamics and optimize tissue perfusion (31).

#### Fluid Underload

Decrease in total body fluid, resulting in fluid deficit of the extracellular and/or intracellular fluid (67). The opposite of *fluid overload*.

#### Goal-Directed Therapy (GDT)

The use of advanced non-invasive and invasive monitoring techniques in conjunction with intravenous fluids, vasopressors or inotropes with the goal of maintaining or establishing hemodynamic stability, adequate tissue perfusion and oxygen delivery to tissues (68, 69).

See *early goal-directed therapy*.

#### Hemodynamic Coherence

Coherence between macrocirculatory and microcirculatory hemodynamics such that regional and microcirculatory perfusion and tissue oxygen delivery permits normal cellular function in support of organ function (70, 71).

#### Hemoglobin-Based Oxygen Carrier (HBOC)

A cell-free hemoglobin solution used in veterinary medicine in the 1990's and 2000's as a blood transfusion substitute to improve oxygen carrying capacity (72–74). HBOC's solutions are not commercially available in the US. Synonym: *oxygen therapeutics*.

#### Hyperchloremic Metabolic Acidosis

Hyperchloremia accompanied by hypobicarbonatemia and metabolic acidosis. Two different mechanistic explanations are bicarbonate loss or dilution (Henderson-Hasselbalch approach) (13) or a decrease in SID caused by an increase in chloride (i.e., 0.9% Na^+^Cl^−^) (4, 13, 75). Synonym: *normal anion gap acidosis*.

#### Hyperoncotic Colloid

A colloid solution with an oncotic pressure above that of plasma (e.g., 10% hydroxyethyl starch, 20% human albumin) (76).

#### Hyperperfusion

Increased (supraphysiologic) blood flow to the tissues (35).

#### Hypertonic Crystalloid

A crystalloid solution with a higher effective osmolality than plasma (e.g., 7.2% sodium chloride: 2464 mOsm/L) (23).

#### Hypertonic Saline (HS)

A sterile hypertonic intravenous crystalloid composed of water, sodium and chloride. Available in multiple concentrations including 3%, 5% and 7.2% (77, 78).

#### Hypertonic-Hyperoncotic Solution

A resuscitation fluid containing a hypertonic crystalloid (>310 mosmol) and a hyperoncotic (>5%) colloid that is used as an alternative small volume fluid resuscitation strategy to rapidly increase intravascular fluid volume in the treatment of hypovolemia [e.g., 7.5% saline and 6% Dextran-70 (HSD)] (79, 80). Synonym: *turbostarch*.

#### Hypervolemia

Excessive circulating blood volume (52, 61). See also *fluid overload*.

#### Hypooncotic Colloid

A colloid solution with an oncotic pressure below that of plasma (e.g., 4% human albumin) (76).

#### Hypoperfusion

Insufficient blood flow to the tissues, resulting in decreased oxygen delivery. End-organ hypoperfusion can manifest as cool extremities, reduced pulse quality, oliguria and tachycardia (81, 82).

#### Hypotensive Resuscitation

See *permissive hypotension*.

#### Hypotonic Crystalloid

A crystalloid solution with a lower effective osmolality than plasma (e.g. 0.45% sodium chloride: 154 mOsm/L) (23). A solution of 5% dextrose in water is also classified as a hypotonic fluid despite having an osmolality (278 mOsm/L) that is similar to plasma, since the dextrose is rapidly taken up into cells and metabolized following infusion, leaving water behind (48).

#### Hypovolemia

Insufficient intravascular fluid volume, which may be absolute such as from dehydration and hemorrhage, or relative such as with vasodilatory shock (61, 83).

#### Ineffective Osmoles

Small dissolved particles in solution that contribute to total osmolality but do not exert an osmotic pressure because they freely cross and equilibrate across cell membranes (e.g., urea, dextrose) (84).

#### Insensible Water Loss

Body fluid losses that cannot be easily measured, such as evaporative losses from the skin and respiratory tract, and the water content of stool (85, 86).

#### Interstitial Fluid

The total volume of extracellular fluid contained within the interstitial tissues surrounding cells (12–15% of total body weight) (55).

#### Intracellular Fluid

The total volume of fluid contained within cells (40% of total body weight) (55).

#### Intravascular Fluid

The total volume of extracellular fluid contained within arteries, veins and capillaries in the circulatory system (6–8% of total body weight) (55).

#### Intravascular Volume Depletion

Reduction in intravascular fluid volume, which is a type of extracellular fluid volume depletion (87). Related term: *volume depletion*.

#### Isooncotic Colloid

A colloid solution with an oncotic pressure similar to that of plasma (e.g., 6% hydroxyethyl starch, 5% human albumin) (88).

#### Isotonic Crystalloid

A crystalloid solution with an osmolality similar to plasma. The two types of isotonic crystalloids are isotonic saline (0.9% sodium chloride) and balanced solutions (e.g., lactated Ringer's, Normosol®-R) (13).

#### Isovolemic Hemodilution

See *acute normovolemic hemodilution*.

#### Lactic Acidosis

Hyperlactatemia with concurrent metabolic acidosis (89–93). Lactate is produced by skeletal muscle and other tissues in large amounts during anaerobic conditions and is commonly used as a marker of the adequacy of tissue perfusion (Type A lactic acidosis). Increased production of L-lactic acid in the absence of hypoxia or increased demand for ATP is termed Type B lactic acidosis. Notably, rapid intravenous administration of lactated Ringer's solution increases plasma lactate concentration within 10 min but baseline values are reestablished within 60 min after cessation of administration (93).

#### Lean Body Mass

Total body weight minus the weight of fat. Calculated as 80% of ideal body weight when 20% of total body weight is fat (94).

#### Liberal vs. Restrictive Fluid Therapy

Term applied to randomized trials investigating the effect on morbidity and mortality of a conservative (restrictive) fluid strategy, compared to a standard (liberal) fluid regimen. Many standard fluid regimens are more likely to result in a positive fluid balance (95–97).

#### Macrocirculation

Large and medium-sized arteries and veins that serve as conduit vessels, transporting blood to and from organs and tissues (98).

#### Maintenance Fluid

A type of crystalloid solution that is designed to maintain hydration by meeting daily water and electrolyte requirements (7, 23, 86). Both hypotonic and isotonic fluids can fulfill these requirements (86).

#### Maintenance Fluid Therapy

A fluid therapy plan designed to provide water and electrolytes in quantities that meet normal daily fluid needs and replace urinary, gastrointestinal, and evaporative losses (86).

#### Massive Hemorrhage

Loss of > 40–50% of total blood volume over 3 h or less, or > 100% of total blood volume over 24 h (99).

#### Massive Transfusion

Replacement of > 40-50% of total blood volume over 3 h (11, 99), or > 100% of total blood volume over 24 h (100, 101).

#### Microcirculation

Blood vessels <200–300 micrometers in diameter, consisting of small arteries, arterioles, capillaries, and venules (98, 102–104).

#### Normal Anion Gap Metabolic Acidosis

See *Hyperchloremic metabolic acidosis*.

#### Normal Saline (NS)

See i*sotonic saline*.

#### Normovolemia

See *euvolemia*.

#### Oncotic Pressure

See *colloid osmotic pressure*.

#### Osmolality

A measure of the concentration of osmotically active particles per unit volume of solution, measured in milliosmoles per liter of solution (mOsm/L) (7, 22, 105). In clinical practice, osmolarity, and osmolality are similar enough to be used interchangeably (106).

#### Osmolarity

A measure of the concentration of osmotically active particles per unit mass of solution, measured in milliosmoles per kilogram of solution (mOsm/kg) (7, 22, 105). In clinical practice, osmolarity, and osmolality are similar enough to be used interchangeably (106).

#### Osmosis

The process by which molecules of a solvent tend to pass through a semipermeable membrane from a less concentrated solution into a more concentrated solution, thus equalizing the concentrations on both sides of the membrane (20).

#### Parenteral

Administration of food or medication through a non-enteral (e.g., non-oral) route, such as intravenous, subcutaneous, intramuscular and intradermal (107).

#### Parkland Formula

A fluid resuscitation protocol for burn patients which calls for lactated Ringer's solution dosed at 4 ml/kg/%TBSA, where %TBSA refers to the percentage of the total body surface area burned (108, 109). Half of the volume is delivered over the first 8 h and the remainder over the next 16 h.

#### Perfusion

The passage of fluid through the circulatory system to organs and tissues (103, 110).

#### Permissive Hypotension

A fluid therapy technique that aims to provide enough resuscitation fluid to ensure adequate end-organ perfusion while maintaining mild hypotension (systolic pressures <90 mmHg) until definitive hemorrhage control can be achieved (32, 33, 111). Synonym: *hypotensive resuscitation*.

#### Plasma

The liquid portion of blood that remains after the cells are removed. Plasma is retrieved by centrifugation of an anticoagulated blood sample, and so unlike serum, it contains fibrinogen and clotting factors (112).

#### Pleth Variability Index (PVI)

An automatic measure of the dynamic change in perfusion index (PI), as determined by a pulse oximeter, occuring during a complete respiratory cycle. The pulse oximeter derived pulsatile signal is indexed against the non-pulsatile infrared signal and expressed as a percentage [PI = (AC/DC) × 100] reflecting the amplitude of the pulse oximeter waveform. The pulse variability index is calculated as PVI = [(PImax – PImin)/PImax] × 100 (113).

#### Polyionic Crystalloid Solution

See *balanced crystalloid solution*.

#### Proteid

A complex biomolecule predominantly made of polypeptides that is found in all living matter (114).

#### Pulse Pressure (PP)

The difference between arterial systolic and diastolic blood pressure measured in millimeters of mercury (mm Hg) (115).

#### Pulse Pressure Variation (PPV)

The difference between the maximum (PPmax) and minimum (PPmin) arterial pulse pressures during one respiratory cycle, divided by their sum divided by 2 ([PPmax + PPmin]/2) and expressed as a percentage (116). Pulse pressure variation is used as a predictor of fluid responsiveness in mechanically ventilated patients.

#### Pulse Wave Transit Time (PWTT)

Time needed for a pulse wave to travel between two arterial sites (117). Due to its inverse relationship to stroke volume, it can be used to assess changes in cardiac output and to evaluate fluid responsiveness in dogs after a fluid challenge (117). It also serves as a marker of arterial stiffness in humans with coronary artery disease (118). PWTT can be calculated as the time from the peak of the R-wave on an electrocardiogram to the rise point of the pulse oximeter wave (the point where it attains 30% of its maximal amplitude) (119).

#### Rebound Hypovolemia

Hypovolemia produced by diuresis induced by rapid bolus fluid administration to conscious animals (120).

#### Relative Hypovolemia

A reduction in the effective circulating blood volume due to venodilation and increased venous capacitance (2). Relative hypovolemia can be caused by drug toxicity (e.g., sensitivity to anesthetic drugs or anesthetic overdose), impairment or loss of compensatory mechanisms, coexisting or induced metabolic or respiratory acidosis, traumatic or surgically induced inflammation, sepsis, cardiogenic shock, and hypothermia.

#### Replacement Fluid

A type of crystalloid solution that is isotonic, has a composition similar to extracellular fluid, and is administered to replace water and electrolyte losses (e.g., Normosol-R, lactated Ringer's, Plasma-Lyte 148) (7, 23).

#### Rescue, Optimization, Stabilization, and Evacuation (ROSE)

A conceptual framework that describes four different stages of fluid resuscitation, beginning with initial rapid fluid administration to treat life-threatening shock (Rescue), continued fluid therapy until adequate perfusion is restored (Optimization), followed by ongoing maintenance fluids (Stabilization), and gradual discontinuation of fluid support (i.e., Evacuation or De-escalation) (31, 109, 121).

#### Resuscitation

See *fluid resuscitation*.

#### Revised Starling Equation

An updated version of the traditional Starling equation that incorporates current understanding of the role of the endothelial glycocalyx in transvascular fluid filtration (44, 122, 123). Related term: *Starling Principle*.

#### Sensible Water Loss

Measurable body fluid losses, such as urine, vomit, and diarrhea (85, 86).

#### Shock

A life-threatening, generalized form of acute circulatory failure associated with inadequate oxygen utilization by the cells, resulting in cellular dysfunction (124). Shock is most commonly organized into four major classifications that have different pathophysiological mechanisms: *hypovolemic shock* refers to reduced effective circulating volume, from internal or external intravascular fluid loss (125); *obstructive shock* results from physical impairment to blood flow, such as from thromboembolic disease; *distributive shock* is caused by maldistribution of blood flow due to loss of vasomotor tone, such as during sepsis or anaphylaxis (124); c*ardiogenic shock* describes cardiac pump dysfunction resulting in decreased forward flow (124, 126).

#### Shock Index (SI)

Calculated as the ratio of the heart rate (HR) divided by systolic blood pressure (SBP) (127). SI >1.0 is a predictor of increased risk of mortality and other markers of morbidity (128).

#### Skin Turgor

The relative elasticity of the skin, used as an estimate of hydration status (129, 130). Skin turgor is evaluated by tenting the skin with the fingers and timing its return to its normal form. Dehydration and increasing age are associated with decreased skin turgor (34).

#### Standard Base Excess (SBE)

The amount of strong acid (millimoles per liter) that needs to be added *in vitro* to 1 L of fully oxygenated whole blood to return the sample to standard conditions (pH of 7.40, PCO_2_ of 40 mm Hg, and temperature of 37°C), at a hemoglobin concentration of ~50 g/L (14, 131). Unlike base excess, standard base excess has been adjusted to reflect the extracellular fluid buffering capacity of hemoglobin *in vivo*. It is used clinically to determine the degree of metabolic acidosis.

Synonym: *base excess of the extracellular fluid (BE*_*Ecf*_*)*. Related term: *base excess*.

#### Starling Principle

Fluid flux across the capillary wall is determined by a balance of hydrostatic and oncotic pressures such that fluid leaves the capillary at the arterial end of the capillary and is absorbed at the venous end of capillary (123).

#### Stressed Vascular Volume (Vs)

The volume of blood within a vein that produces a transmural pressure above zero (52, 132). Compare to *unstressed vascular volume*.

#### Stroke Volume Variation (SVV)

The difference between maximum and minimum stroke volume during one respiratory cycle, divided by their mean, and averaged over several breaths (133). Stroke volume variation is used as an indicator of fluid responsiveness in mechanically ventilated patients.

#### Strong Ion

A cation and anion that are considered to be fully dissociated at physiologic pH (13, 131, 134, 135). The major strong cations in plasma are sodium, calcium and magnesium, while the major strong anions are chloride, sulfate, and lactate.

#### Strong Ion Difference (SID)

The difference between the concentrations of strong cations and strong anions in plasma (134–136).

#### Strong Ion Gap (SIG)

SIG quantifies [unmeasured anions] – [unmeasured cations] of both strong and weak ions. It is reflects the difference between the activity of all common cations (Na^+^, K^+^, Mg^2+^, Ca^2+^) with the common anions (Cl^−^, lactate, urate) and other measured non-volatile weak acids (A^−^). SIG is calculated as SIDa – SIDe, or more specifically, as [Na^+^] + [K^+^] + [Mg^2+^] + [Ca^2+^] – [Cl^−^ corrected] – [lactate] - [A^−^] – [${\text{HCO}}_{3}^{-}$], in milliequivalents per liter; where SIDa is the apparent strong ion difference and SIDe is the effective strong ion difference (137). An increased SIG is a predictor of mortality (8).

#### Third Spacing

The pathological shift of fluid into extracellular sites in the body that are anatomically separated from other body fluid compartments and where the fluid is considered to have no physiological function (138–140). In human patients, the fluid movement is theorized to occur following trauma or major surgery into ill-defined spaces following intravenous fluid administration. However, these “spaces” have since been identified to include interstitial fluids. Third space fluids are eventually reabsorbed into the central fluid compartment and are therefore considered to be a myth (139, 140). In veterinary medicine, the term is also used to refer to the loss of fluids into body cavities, such as the pleural space, peritoneal space and gastrointestinal lumen (141).

#### Titration

See *fluid titration*.

#### Tonicity

A measurement of the effective osmolality of a solution, which corresponds to its ability to cause water to diffuse across a semi-permeable membrane, such as the cell membrane (20, 142–144). A cell will swell when placed in a hypotonic solution, shrink when placed in a hypertonic solution, and have an unchanged volume in an isotonic solution.

#### Total Body Water

The total water content in the body, which represents the sum of the intracellular and extracellular fluid volumes (43, 52). Total body water is ~60% of ideal body weight in adult dogs (55), cats (55), and horses (145).

#### Unstressed Vascular Volume (Vu)

The volume of blood in a vein that produces a transmural pressure equal to zero (52, 132). The sum of the stressed (~30% of total volume) and unstressed (~70% of total volume) volumes is the total blood volume within the venous system (132). Compare to *stressed vascular volume*.

#### Vascular Hyporesponsiveness

A decreased vascular response to fluid therapy or the pressor effects of exogenous vasopressors (146). Vascular reactivity to the administration of catecholamines or other vasopressors (e.g., vasopressin) is used to predict mortality (146, 147).

#### Vascular Volume

See *blood volume*.

#### Vasoplegia

Severely low systemic vascular resistance in conjunction with profound hypotension and a normal or increased cardiac output (148). Occasionally referred to as vasoplegic shock and used synonymously with distributive shock, the vasoplegic syndrome may occur in septic shock, after surgery, burns, severe pancreatitis or extensive trauma (148).

#### Volume Depletion

The loss of water and electrolytes from the extracellular fluid compartment (34). Volume depletion can be classified by the location of the lost fluid, such as intracellular dehydration or extracellular fluid loss. It can also be characterized by the salt and water content of the fluid loss. *Hypotonic fluid loss*: A predominantly pure water deficit which is caused by water loss exceeding solute loss in the extracellular fluid, or by insufficient water intake relative to water output, leading to hypernatremic volume depletion. *Hypertonic fluid loss*: A deficit of water and solutes from the extracellular fluid, where the solute loss exceeds water loss, resulting in the development of hypoosmolality of the extracellular fluid and hyponatremic volume depletion. *Isotonic fluid loss*: A proportionate deficit of water and solutes from the extracellular fluid, therefore the osmolality of the extracellular fluid does not increase.

#### Volume Kinetics

The study of how water is distributed and eliminated following an infusion of intravenous fluids (149, 150).

#### Volume Overload

See *fluid overload*.

#### Zero Balance Fluid Therapy

A restrictive regimen aiming to avoid postoperative fluid retention (as indicated by weight gain) (151, 152).

### Routes of Fluid Administration

Fluids are typically administered to veterinary patients through enteral, subcutaneous and intravenous routes (23, 48, 153), or less commonly, into the medullary cavity (154) or into the coelom in reptiles (155). The ideal method of fluid delivery will vary depending on the species, the underlying disease processes, and the size of the fluid deficit. Animals that require rapid correction of life threatening conditions, such as hypovolemic shock, benefit from the rapid intravascular volume expansion achievable with intravenous fluids. On the other hand, non-intravenous fluid delivery routes, which are characterized by slower rates of absorption (156), may be sufficient in stable patients to meet maintenance fluid needs, treat lesser degrees of dehydration or keep up with abnormal ongoing losses.

Subcutaneous or enteral fluids can be considered in small animal patients with mild to moderate fluid deficits. Compared to intravenous fluid administration, there is a lower limit to the amount of fluid that can be delivered by these routes. In the presence of severe dehydration and hypovolemia, avoid fluid replacement by the subcutaneous route due to the potential for decreased absorption resulting from peripheral vasoconstriction (7, 48).

Where larger volumes of fluid are necessary but venous access cannot be obtained (a common dilemma in many pediatric, avian, and exotic animals), placement of an intraosseous (IO) catheter may be considered (154, 157). Despite increased resistance to flow compared to intravenous infusion, reasonably high fluid administration rates can still be achieved with intramedullary delivery (158). The gravity-dependent rate of fluid delivery was greater in the femur and humerus compared to the tibia and ilium in one canine cadaveric study (159).

When voluntary oral intake is insufficient or undesirable, fluids can be rapidly and inexpensively administered by nasogastric tube to horses (153, 160) and ruminants (161, 162) to treat mild to moderate dehydration. In some cases, parenteral methods may be preferred in camelids to reduce stress (163). As with companion animal species, intravenous fluids are preferred when severe dehydration is present due to the potential for reduced bowel absorption resulting from hypoperfusion (153).

Alternative fluid administration sites have also been described. A 6-h crystalloid infusion given per rectum was reported to be safe and well-tolerated in horses (156), although the usefulness of this technique in clinical settings remains undetermined. Unlike in reptiles, where intracoelomic fluid delivery is well-described (155), intraperitoneal fluid administration is rarely discussed or performed in other species (48) due the presence of safer alternatives.

## Discussion

Despite the frequency with which fluids are administered to veterinary patients, developing an effective fluid management plan may at times be surprisingly complex. A thorough understanding of the physiology of body fluids, fluid administration routes, therapeutic delivery strategies, risks, and complications will help to optimize patient outcomes. National and multinational organizations, such as the International Fluid Academy (IFA), provide opportunities for clinicians and researchers to promote research and education in the practice of fluid therapy. The use of clear and consistent terminology is a key component to fostering effective communication and collaboration within the veterinary and human healthcare fields.

## Author Contributions

The author confirms being the sole contributor of this work and has approved it for publication.

## Conflict of Interest

The author declares that the research was conducted in the absence of any commercial or financial relationships that could be construed as a potential conflict of interest.
